# Phylogenetic group determination and plasmid virulence gene profiles of colistin-resistant *Escherichia coli* originated from the broiler meat supply chain in Bogor, Indonesia

**DOI:** 10.14202/vetworld.2020.1807-1814

**Published:** 2020-09-05

**Authors:** Irma Rahayuningtyas, Agustin Indrawati, I Wayan Teguh Wibawan, Maria Fatima Palupi, Istiyaningsih Istiyaningsih

**Affiliations:** 1 Department of Animal Disease and Veterinary Public Health, Faculty of Veterinary Medicine, IPB University-Bogor, Indonesia; 2 National Veterinary Drug Assay Laboratory, Directorate General of Livestock and Animal Health, Ministry of Agriculture of the Republic of Indonesia, Indonesia

**Keywords:** broiler supply chain, *Escherichia coli*, phylogenetic group, virulence gene

## Abstract

**Background and Aim:**

Pathogenic *Escherichia coli* contamination along the broiler meat supply chain is a serious public health concern. This bacterial infection with multidrug-resistant can lead to treatment failure. Several studies have revealed that avian pathogenic *E. coli* (APEC) and human extraintestinal pathogenic *E. coli* (ExPEC) showed a close genetic relationship and may share virulence genes. This study aimed to determine the phylogenetic group and virulence gene profiles in colistin-resistant *E. coli* obtained from the broiler meat supply chain in Bogor, West Java, Indonesia.

**Materials and Methods:**

Fifty-eight archive isolates originated from the cloacal swab, litter, drinking water, inside plucker swab, fresh meat at small scale poultry slaughterhouses, and traditional markets were used in this study. All the isolates were characterized by a polymerase chain reaction to determine the phylogenetic group (A, B1, B2, or D) and virulence gene profiles with APEC marker genes (*iutA*, *hlyF*, *iss*, *iroN*, and *ompT*).

**Results:**

Phylogenetic grouping revealed that the isolates belong to A group (34.48%), D group (34.48%), B1 group (17.24%), and B2 group (13.79%). The virulence gene prevalence was as follows: *iutA* (36%), *hlyF* (21%), *ompT* (21%), *iroN* (10%), and *iss* (9%). The B2 group presented with more virulence genes combinations. *iroN*, *hlyF*, and *ompT* genes were positively associated with the B2 group (p≤0.05).

**Conclusion:**

Our results highlight the role of colistin-resistant *E. coli* originated from the broiler meat supply chain as a potential reservoir for human ExPEC virulence genes.

## Introduction

Most *Escherichia coli* are commensal bacteria, but some strains are pathogenic in humans and warm-blooded animals. One of the critical pathogenic *E. coli* is extraintestinal pathogenic *E. coli* (ExPEC). This pathotype may cause systemic infections in humans and animals. ExPEC subpathotypes include uropathogenic *E. coli* (UPEC), neonatal meningitis *E. coli* (NMEC), sepsis-associated *E. coli* (SePEC), and avian pathogenic *E. coli* (APEC). UPEC causes urinary tract infections (UTI), NMEC leading to neonatal meningitis, and SePEC, which causes sepsis in humans [1]. APEC is the causative agent of colibacillosis, which may cause economic losses to the worldwide poultry industry [2]. Colibacillosis signs are airsacculitis, polyserositis, coligranuloma, cellulitis, omphalitis, swollen head syndrome, and systemic infections [3].

Several studies reported that there are genetic similarities between ExPEC in chickens and humans, especially in virulence genes [4,5]. Plasmids that carry virulence genes in APEC can be a source of virulence genes for commensal *E. coli* strains or other human ExPEC [6,7]. This statement is consistent with the hypothesis that poultry products can act as reservoirs of potentially zoonotic pathogenic bacteria [8]. Conversely, several studies have proven that isolates that cause ExPEC in humans may cause colibacillosis in chicken and laboratory animals [9,10]. The problem increases when virulence genes and antibiotic resistance genes can be transferred to other bacteria, both commensal and pathogenic [6-8]. This problem causes the need for research on the potency of colistin-resistant *E. coli* originated from the broiler meat supply chain as a reservoir of colistin-resistance genes and ExPEC virulence genes in humans.

This study was conducted to determine the phylogenetic group and virulence gene profiles by multiplex polymerase chain reaction (PCR) method from colistin-resistant *E. coli*, which was originated from the broiler meat supply chain in Bogor, West Java, Indonesia.

## Materials and Methods

### Ethical approval

This study did not use live animals, so ethical approval was not needed.

### Study period and location

The study was conducted from August 2019 to February 2020. Culture and re-identification, phylogenetic group determination, and detection of virulence genes were carried out at the National Veterinary Drug Assay Laboratory (NVDAL), Directorate General of Livestock and Animal Health Services, Ministry of Agriculture of the Republic of Indonesia.

### Samples

The colistin-resistant *E. coli* used in this study was derived from 58 archive isolates isolated in 2017 from the broiler meat supply chain in Bogor, West Java Indonesia [11]. The samples originated from the cloacal swab, litter, drinking water, inside plucker swab, fresh meat at small scale poultry slaughterhouses (SSPS), and traditional markets (TM). All of these isolates were tested for sensitivity to colistin sulfate using the agar dilution method.

### Culture and identification

All isolates were cultured and re-identified. These isolates were inoculated on the heart infusion broth (HIB) media (Difco, USA) and incubated at 37°C for 18-24 h. These bacteria were cultured on MacConkey agar (Oxoid, UK) and eosin methylene blue agar (Oxoid, UK) and incubated at 37°C for 18-24 h. The *E. coli* suspected colony inoculated on heart infusion agar (HIA) media (Difco, USA) for 18-24 h at 37°C, then the Gram staining and biochemical testing were carried out. Biochemical tests used were the IMViC test and triple sugar iron agar (Oxoid, UK). The IMViC test consists of sulfite indole motility (Oxoid, UK), methyl red-Voges–Proskauer (MR-VP) (Merck, Germany), and Simmons citrate agar (Oxoid, UK). Isolates confirmed by *E. coli* showed IMViC results: Indole (+), MR (+), VP (−), and Citrate (−) [12]. *E. coli* ATCC 25922 was used as a positive control. All archive *E. coli* isolates were reconfirmed their sensitivity to colistin sulfate antibiotics using the agar dilution method [13,14].

### Genomic extraction of *E. coli* DNA

The *E. coli* isolates were enriched with HIB for 18-24 h at 37°C. The isolates then inoculated on HIA for 18-24 h at 37°C. *E. coli* colonies on HIA were further extracted for phylogenetic group determination, as well as the detection of virulence genes. DNA extraction using the boiling method with BufferPrepMan^®^ Ultra Sample Preparation Reagent (Applied Biosystems, USA).

### Phylogenetic group determination with PCR

The determination of *E. coli* was carried out according to the method of Clermont *et al*. [15] to the A, B1, B2, or D phylogenetic groups. This method is based on the analysis of the presence of the *chuA* and the *yjaA* genes also DNA fragments (TSPE4.C2) by the triplex PCR method. Primer sequences, gene targets, amplicon lengths, and annealing temperatures are shown in Table-1 [15]. DNA amplification using the HotStarTaq^®^ Master Mix Kit (Qiagen, Germany) with a total PCR reagent volume of 25 μL consisting of 12.5 μL HotStarTaq^®^ Master Mix, 1 μL of each primer (20 μM), 5.5 μL dH_2_O, and 5 μL DNA templates. The PCR process using Proflex™ 3×32-well PCR System (Thermo Fisher Scientific, USA). The PCR was carried out with a 95°C predenaturation cycle for 15 min, followed by 30 cycles consisting of denaturation of 94°C for 1 min, annealing 54°C for 1 min, and extension at 72°C for 1 min. The final extension was carried out at 72°C for 1 min. PCR products were visualized using a 1.5% agarose gel with SYBR Safe DNA Gel Stain alongside a 100 bp DNA Ladder (Thermo Fisher Scientific, USA). *E. coli* ATCC 25922 was used as a positive control of the *chuA*, *yjaA* genes, and DNA fragments (TSPE4.C2) [16] together with *E. coli* APEC strain O2 [17] obtained from the NVDAL.

**Table-1:** Primer sets for phylogenetic group determination of *Escherichia coli.*

No.	Target gene (group)	Primer	Annealing temperature (°C)	Amplicon (bp)	References
1	*chu*A (B2 or D)	(F) 5′- GACGAACCAACGGTCAGGAT-3′	54	279	[15]
		(R) 5′- TGCCGCCAGTACCAAAGACA-3′			
2	*yja*A (B2)	(F) 5′- TGAAGTGTCAGGAGACGCTG-3′	54	211	
		(R) 5′- ATGGAGAATGCGTTCCTCAAC-3′			
3	TspE4C2 (B1 or A)	(F) 5′- GAGTAATGTCGGGGCATTCA-3′	54	152	
		(R) 5′- CGCGCCAACAAAGTATTACG-3′			

### Detection of virulence genes by PCR

The detection of virulence genes was carried out according to the method of Johnson *et al*. [18] targeting *iutA*, *hlyF*, *iss*, *iroN*, and *ompT* genes. Primary sequences, gene targets, amplicons, and annealing temperatures are shown in Table-2 [18-21]. DNA amplification using the HotStarTaq^®^ Master Mix Kit (Qiagen, Germany) with a total PCR reagent volume of 25 μL consisting of 12.5 μL HotStarTaq^®^ Master Mix, 1 μL of each primer (20 μM), 5.5 μL dH_2_O, and 5 μL DNA templates. The PCR process using Proflex™ ×32-well PCR System (Thermo Fisher Scientific, USA). The PCR process was carried out with a 95°C predenaturation cycle for 15 min. The next process is 30 cycles consisting of denaturation of 94°C for 1 min, annealing 63°C for 1 min, and extension at 72°C for 1 min, and the end of the amplification is followed by a final extension at 72°C for 1 min. PCR products were visualized on a 1.5% agarose gel with SYBR Safe DNA Gel Stain and using 100 bp DNA Ladder (Thermo Fisher Scientific, USA). Positive control of the five APEC marker genes used *E. coli* O2 strain obtained from the NVDAL.

**Table-2:** Primer sets for detection virulence gene profiles of *Escherichia coli.*

No.	Target gene	Primer	Annealing temperature (°C)	Amplicon (bp)	References
1	*iroN*	(F) 5′- AATCCGGCAAAGAGACGAACCGCCT -3′	63	553	[19]
		(R) 5′- GTTCGGGCAACCCCTGCTTTGACTTT -3′			
2	*ompT*	(F) 5′- TCATCCCGGAAGCCTCCCTCACTACTAT-3′	63	496	[19]
		(R) 5′- TAGCGTTTGCTGCACTGGCTTCTGATAC -3′			
3	*hlyF*	(F) 5′- GGCCACAGTCGTTTAGGGTGCTTACC -3′	63	450	[20]
		(R) 5′- GGCGGTTTAGGCATTCCGATACTCAG -3′			
4	*iss*	(F) 5′- CAGCAACCCGAACCACTTGATG-3′	63	323	[21]
		(R) 5′- AGCATTGCCAGAGCGGCAGAA- 3′			
5	*iutA*	(F) 5′- GGCTGGACATCATGGGAACTGG -3′	63	302	[18]
		(R) 5′- CGTCGGGAACGGGTAGAATCG -3′			

### Statistical analysis

The distribution pattern of phylogenetic groups and virulence genes was compared with Fisher’s exact test (two-sided) with a confidence value of p<0.05. This statistical test was performed with the SPSS statistical program version 2.3 (IBM Corp., NY, USA).

## Results

**Figure-1:**
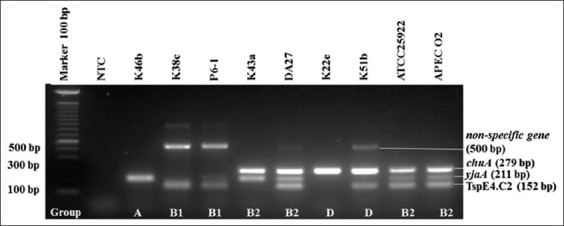
Triplex polymerase chain reaction phylogenetic group determination of *Escherichia coli*. NTC: Non-template control; isolate code: K46b (Group A); K38c and P6-1 (Group B1); K43a and DA27 (Group B2); K22e and K51b (Group D); positive control: *E. coli* ATCC 25922 and avian pathogenic *E. coli* O2 strain (Group B2).

The results of the re-identification of all 58 isolates of colistin-resistant *E. coli* archive showed that all isolates were confirmed to be *E. coli*. All of the isolates were resistant to colistin sulfate with a minimum inhibitory concentration value of more than 2 μg/μL [13]. Phylogenetic determination of isolates carried out by multiplex PCR (Figure-1) showed that 34.48% isolates belong to Group A (20/58), 34.48% isolates were Group D), 17.24% were Group B1 (10/58), and 13.79 % belong to Group B2 (8/58) (Table-3). The combination of three genetic markers, namely, the *chuA* and *yjaA* genes, also the TSPE4.C2 fragment, can distinguish the *E. coli* phylogenetic groups. *E. coli* ATCC 25922 and APEC O2 strain were used as positive controls. Both of them showed bands from the three genetic markers.

**Table-3:** Phylogenetic group determination of colistin-resistant *Escherichia coli* along broiler meat supply chain in Bogor.

Phylo group	Genetic marker			Type of isolate source						Total (%)
	
	*chuA*	*yjaA*	TSPE4.C2	Cloacal swab	Drinking water	Litter	Inside plucker swab	Fresh meat (SSPS)	Fresh meat (TM)	
A	-	±	-	9/31	2/4	1/4	0/2	3/7	5/10	20/58 (34.48)
B1	-	±	+	5/31	2/4	0/4	1/2	1/7	1/10	10/58 (17.24)
B2	+	+	±	5/31	0/4	1/4	0/2	0/7	2/10	8/58 (13.79)
D	+	-	±	12/31	0/4	2/4	1/2	3/7	2/10	20/58 (34.48)
Total				31	4	4	2	7	10	58

SPSS=Small scale poultry slaughterhouse, TM=Traditional market

The distribution patterns of phylogenetic groups and virulence genes in 58 isolates of colistin-resistant *E. coli* are shown in Table-4. The *iutA* gene was the most prevalent gene as it was detected in 21 isolates (36%), followed by the *hlyF* gene and the *ompT* gene, 12 isolates each (21%), the *iroN* gene, six isolates (10%), and the *iss* gene, five isolates (9%). The isolates included in the B2 phylogenetic group had more virulence genes, indicated by an average virulence gene (virulence score) of 2.2, followed by Group D with a score of 1.3, then Groups A and B1 with a score of 0.4 each, respectively. The *iroN*, *hlyF*, and *ompT* genes had a positive association with the phylogenetic Group B2 (p≤0.05).

**Table-4:** Phylogenetic group and virulence gene profiles of 58 colistin-resistant *Escherichia coli* isolate.

Group (n)	Prevalence n (%)					

	*iroN*	*hlyF*	*iutA*	*ompT*	*iss*	VS^#^
A (20)	1 (5)	1 (5)	4 (20)	1 (5)	1 (5)	0.4
B1 (10)	1 (10)	1 (10)	0 (0)	1 (10)	1 (10)	0.4
B2 (8)	3 (37)*	4 (50)*	5 (62)	4 (50)*	2 (25)	2.2^#^
D (20)	1 (5)	6 (30)	12 (60)	6 (30)	1 (5)	1.3
Total (58)	6 (10)	12 (21)	21 (36)	12 (21)	5 (9)	1.0

* The positive association between phylogenetic group and virulence genes (p≤0.05).

# VS=Virulence score. A virulence score was calculated for each isolate as the sum of all virulence genes. p value was determined using Fisher’s exact test for comparisons among phylogenetic groups and virulence genes

**Figure-2:**
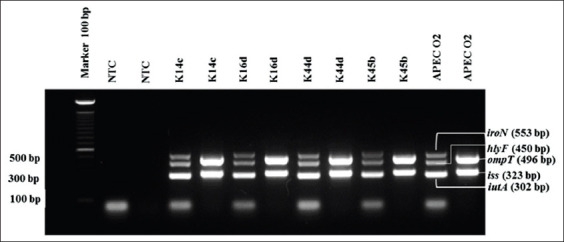
Multiplex polymerase chain reaction profile from five genetic markers (*iroN, hlyF, iutA, ompT*, and *iss*) can detect avian pathogenic *Escherichia coli* (APEC) strains. Isolate code K14c, K16d, K44d, and K45b showed positive results of *iroN, hlyF, iutA, ompT*, and *iss*. NTC: Non-template control; positive control: *E. coli* APEC O2.

The combination of five APEC virulence genes can be detected in isolates from the phylogenetic Group B2 (3.45%), followed by Groups D and A (1.72%). Thirty-six isolates did not express the five virulent genes (Table-5). The multiplex PCR profile of the five APEC genetic markers, namely, *iroN*, *hlyF*, *iutA*, *ompT*, and *iss* genes, is shown in Figure-2. The multiplex PCR results were able to show that the four isolates (K14c, K16d, K44d, and K45b) were APEC (Table-5). Positive control of *E. coli* APEC strain O2 showed positive results on these five APEC markers.

**Table-5:** Combined profile of virulence genes and phylogenetic groups in 58 *Escherichia coli* isolates.

Phylogroup (n)	Combined profile of virulence genes (%)					Not detected

	*iroN, hlyF, iutA, ompT, iss*	*iroN, hlyF, ompT, iss*	*hlyF, iutA, ompT*	*iroN, iutA*	*iutA*	
A (20)	1 (1.72)	0 (0)	0 (0)	0 (0)	3 (5.17)	16 (27.59)
B1 (10)	0 (0)	1 (1.72)	0 (0)	0 (0)	0 (0)	9 (15.52)
B2 (8)	2 (3.45)	0 (0)	1 (1.72)	1 (1.72)	0 (0)	3 (5.17)
D (20)	1 (1.72)	0 (0)	6 (10.34)	0 (0)	6 (10.34)	8(13.79)
Total (58)	4 (6.90)	1 (1.72)	7 (12.07)	1 (1.72)	9 (15.52)	36 (62.07)

Virulence genes: *iroN, hlyF, iutA, ompT,* and *iss*

## Discussion

This study provides the results of the determination of phylogenetic groups and virulence gene profiles of colistin-resistant *E. coli* originated from the broiler meat supply chain in Bogor, West Java, Indonesia. *E. coli* can be divided into pathogenic and commensal. Commensal *E. coli* was the majority strain and designated as a nonpathogenic strain. APEC, subpathotype of ExPEC, has the potential to cause infection in humans, vice versa. It is suggesting that these strains may lack host specificity [22]. APEC, UPEC, and NMEC have overlapping virulence encoding genes [9,23]. This APEC virulence gene can support the ExPEC virulence combination in survival, colonization, and infection. Therefore, APEC isolates have a potential zoonosis risk. Because of some virulence genes commonly found in mobile genetic elements like plasmid, there is a possibility of transfer of virulence genes to other commensal and pathogenic *E. coli* strains [18]. Apart from being a reservoir of antibiotic-resistant genes, APEC may act as a reservoir of virulence genes for ExPEC in humans [7].

The phylogenetic group determination was carried out by multiplex PCR with three genetic markers based on Clermont *et al*. [15] scheme. These markers were chosen because it was more practical and had results that were strongly correlated with other standard methods to distinguish *E. coli* phylogenetic groups [15,24]. As a positive control, *E. coli* ATCC 25922 and APEC O2 strain showed the positive results of the three genetic markers, which indicated that the isolates belong to the B2 group (Figure-1). In PCR results, positive isolates of TspE4.C2 fragments (152 bp) are often, followed by the appearance of non-specific bands with an amplicon of 500 bp. This extra band is seen more clearly in some isolates which belong to the B1 group. These results are consistent with research conducted by Castillo *et al*. [25] that stated that sequencing from the 528 bp extra band showed that there was 100% homology with lipase (acetyl-hydrolase) found in pathogenic strains of *E. coli* belonging to the B1 group. Therefore, this extra band could be used to differentiate pathogenic *E. coli* isolates and commensals included in the B1 group. In this study, out of the ten isolates belonged to the B1 group, four were commensal. The remaining six were pathogenic isolates that were characterized by the presence of extra bands in 500 bp. The analysis of the results of the determination of phylogenetic groups showed that the majority of isolates belong to Groups A and D, followed by Groups B1 and B2. According to Johnson *et al*. [18], ExPEC is mostly included in Groups B2 and D, while non-pathogenic *E. coli* tends to belong to the B1 group and the A group.

The identification of virulence gene profile was carried out by the multiplex PCR method with five plasmid genes as APEC markers (*iss*, *iroN*, *ompT*, *iutA*, and *hlyF* genes) [18]. These five genes are similar to the virulence genes in ExPEC that infect humans. Therefore, the *E. coli* strain with the five genes in the plasmid can potentially be zoonotic [8]. Studies in several countries showed that most APEC strains belong to the B2 group and the D group [17,26,27], as well as ExPEC in the majority of humans, including in the B2 group and several D groups [15,28]. In this study, four APEC isolates were obtained (with code K14c, K16d, K44d, and K45b) (Figure-2). All four isolates, along with positive control of the *E. coli* APEC O2 strain, showed positive PCR results on the five APEC marker genes. Of the four APEC isolates, two of them belong to the B2 group (K14c and K44d), and two of the other isolates belong to the D group (K16d) and the A group (K45b). The presence of A group that is commensal *E. coli* raises the suspicion that the commensal isolate has received all five APEC virulence genes from other *E. coli*. Several studies have stated that commensal *E. coli* can obtain virulence genes through horizontal gene transfer from pathogenic strains that cause intestinal or extraintestinal diseases in animals and humans [4,29]. The most prevalent virulence genes were *iutA* (36%), followed by *hlyF* and *ompT* (21%), *iroN* (10%), and *iss* (9%). The *iutA* gene is a pathogenic gene that causes APEC [30,31]. The study conducted by Johnson *et al*. [19] stated that more than 80% of *E. coli* isolates possessing plasmid genes, namely, the *iroN*, the *iutA*, and the *hlyF* genes, play a role in colibacillosis in poultry. Filho *et al*. [3] reported that 83% of the 994 APEC samples were positive for *iroN* and *hlyF* genes.

The isolates belonging to the B2 phylogenetic group had more virulence factor encoding genes, indicated by the highest virulence scores, followed by the D group, the A group, and the B1 group (Table-4). The *iroN*, *hlyF*, and *ompT* genes have a positive association with the phylogenetic group B2 (P ≤ 0.05). The *hlyF* gene plays a role in the formation of outer membrane vesicles (OMVs) in *E. coli* bacteria and in the preparation of the hemolysin F protein that functions to lyse the host’s red blood cells [32]. Several studies found that the *ompT* gene can break down the host antimicrobial peptides, namely, protamine and plasminogen from *E. coli* isolated from patients with UTI and APEC [33,34]. The *iss* gene (increased serum survival) is related to APEC’s self-defense mechanism against the host’s body’s defense system [18].

The most prevalent APEC virulence gene in this study was *iutA* gene (36%). The *iutA* as well as *iroN* virulence genes are very important for APEC and human ExPEC to maintain their life in the host body. Both of these genes encode siderophore. Siderophore is an iron-binding molecule, which binds the ferric ion in *E. coli* external environment. The *iutA* gene encodes the aerobactin siderophore receptor, while the *iroN* gene encodes salmochelin siderophore [35,36]. The iron itself is a vital component in bacterial biological processes, such as energy formation, oxygen transport, and DNA replication [37]. APEC and human ExPEC develop the mechanism of iron acquisition systems from their host body by producing siderophore. It is because of the low availability of iron in the extraintestinal site in the location where the infection occurs. Recent studies have shown that this system contributes to the virulence of APEC and human ExPEC in terms of attachment and invasion [38,39].

The combination of the APEC virulence genes can be detected in isolates from the phylogroup B2 (3.45%), followed by Groups D and A (1.72%). Isolates included in Groups A and B1 had a combination of APEC virulence factor coding genes (Table-5). Based on these results, there are presumptions that the isolate obtained the acquisition of virulence genes from other APEC isolates. According to Johnson *et al*. [19], the *iroN*, *hlyF*, *iutA*, *ompT*, and *iss* genes are found in the pAPEC-O2-ColV plasmid. This genetic information (virulence genes) can be transferred through horizontal gene transfer mechanisms by mobile plasmids [40]. The undetection of the five virulence genes in 36 isolates showed that the isolates were probably commensal or derived from other pathotypes to have different virulence factor combinations.

It is essential to determine phylogenetic groups and virulence gene profiles in colistin-resistant isolates from the broiler meat supply chain. Five genes studied in this research are APEC markers plasmid genes. These genes may cause colibacillosis in chickens, as well as create zoonotic potential and may act as a reservoir of virulence genes for human ExPEC can be propagated to human and other strains of *E. coli*, both commensal and pathogenic. Besides being useful in the characterization of pathogenic *E. coli* isolates, studies of the profile of virulence genes in *E. coli* are essential because they can be used as a basis for developing effective vaccines [41].

In this study, there was an isolate that originated from fresh meat in TM belongs to the B2 group and have two genes, namely, the *iroN* and the *iutA* genes, located in the plasmid and may act as human ExPEC reservoirs. One isolate belongs to the B1 commensal group originating from the cloacal swab. Still, it has four genes encoding the virulence APEC factor, so there is a suspicion that the isolate obtained the virulence gene acquisition from another APEC isolate. Several studies stated that genetic information, in this case, virulence genes, can be transferred through the mechanism of horizontal gene transfer by mobile genetic elements (plasmids, bacteriophages, and genomic islands). The evolution of *E. coli* strains is highly determined in the genomic region, and this mechanism [5,40,42,43]. The virulence APEC genes and the antibiotic-resistant genes are often on the same plasmid [44], so it potential to be transferred to other *E. coli* bacteria.

*E. coli* isolates with five virulence genes are at risk of causing zoonotic diseases in poultry workers, workers in SSPS, the sellers in TM, and consumers if they consume meat that is not processed correctly. These isolates have a zoonotic risk and also as a reservoir of ExPEC virulent genes in the environment. Healthy chickens can spread *E. coli*, which has virulence genes into the environment, through manures derived from farm waste that can contaminate agricultural fields and public waters [5,45]. The potential spread throughout the broiler meat supply chain must be minimized to produce a product that is safe for consumption. Good farming practices, especially the sanitation of the bedding and proper ventilation system, must be considered. Probiotics and vaccinations are needed to prevent colibacillosis on broiler farms. Hygiene and sanitation at the retail market, as well as proper carcass processing, can reduce the potency for the spread of zoonotic pathogenic *E. coli* and antibiotics resistant [46].

Further research is needed to prove the virulence of APEC isolates in the host. According to Filho *et al*. [3], subcutaneous inoculation of APEC isolates in embryos or day-old chicken (DOC) is the gold standard method for confirming the virulence of *E. coli*. *E. coli* isolates are proven to be pathogenic if the embryo dies or there are specific lesions, followed by death in the DOC. The mechanism of virulence genes transfer horizontally needs to be proven.

## Conclusion

Pathogenic *E. coli* isolates from phylogroup B2 and D are found along the broiler meat supply chain obtain from Bogor, West Java. Virulence factor coding genes are also found in isolates from Groups A and B1 commonly owned by commensal *E. coli*. The B2 group isolates had a positive association with the virulence APEC marker genes contained in the plasmid (*iroN*, *hlyF*, and *ompT*). The virulence genes can potentially be transferred to other *E. coli*, both commensal and pathogenic. The isolate can contaminate carcass if the processing does not conform to proper procedures. It was found to be a potential danger from the spread of virulence genes by *E. coli* isolates obtained from the broiler meat supply chain in Bogor, West JavaIndonesia.

## Authors’ Contributions

IR designed the study and drafted the manuscript under the supervision of AI and IWTW. MFP collected samples and did colistin resistance susceptibility testing. IR performed the test and data analysis under the supervision of AI, IWTW, and II. All authors have read and approved the final manuscript.
